# Signs, Fines and Compliance Officers: A Systematic Review of Strategies for Enforcing Smoke-Free Policy

**DOI:** 10.3390/ijerph15071386

**Published:** 2018-07-02

**Authors:** Olivia Wynne, Ashleigh Guillaumier, Laura Twyman, Sam McCrabb, Alexandra M. J. Denham, Christine Paul, Amanda L. Baker, Billie Bonevski

**Affiliations:** 1 School of Medicine & Public Health, University of Newcastle, University Drive Callaghan, Callaghan, NSW 2308, Australia; olivia.wynne@newcastle.edu.au (O.W.); ashleigh.guillaumier@newcastle.edu.au (A.G.); Laura.Twyman@nswcc.org.au (L.T.); sam.mccrabb@newcastle.edu.au (S.M.); alexandra.denham@newcastle.edu.au (A.M.J.D.); chris.paul@newcastle.edu.au (C.P.); amanda.baker@newcastle.edu.au (A.L.B.); 2 Tobacco Control Unit, Cancer Council NSW, 153 Dowling Street, Woolloomooloo, NSW 2011, Australia

**Keywords:** smoke-free environment, enforcement strategies, smoke-free policy, smoking cessation

## Abstract

*Background*. Smoke-free environment policies limit or eliminate the use of smoke-producing tobacco in designated areas thereby reducing second hand smoke. Enforcement is perceived as critical to the successful adoption of a smoke-free policy. However, there is limited guidance available regarding effective enforcement strategies. A systematic review was conducted to examine the effectiveness of enforcement strategies at increasing compliance with and enforcement of smoke-free policies; and to determine circumstances other than enforcement strategies that are associated with compliance with smoke-free policies. *Design*. Medline, Medline in Process, The Cochrane Library, Embase, PsycInfo and CINAHL databases were searched using MeSH and keywords for relevant studies published between January 1980 and August 2017. A narrative synthesis and methodological quality assessment of included studies was undertaken. *Results*. Policy promotion and awareness-raising activities, signage, enforcement officers, and penalties for violations were the enforcement strategies most frequently cited as being associated with successful policy enforcement. Additionally, awareness of the laws, non-smoking management and lower staff smoking rates, and membership of a network guiding the policy enforcement contributed to higher compliance with smoke-free policies. *Conclusions*. There is weak evidence of the effectiveness of strategies associated with compliance with smoke-free policies. Given the evidence base is weak, well-designed trials utilizing appropriate evaluation designs are needed. Overall enforcement strategies associated with total smoke-free bans resulted in higher levels of compliance than strategies for policies that had only partial smoke-free bans.

## 1. Introduction

There is strong evidence that inhaling other people’s tobacco smoke is harmful to health. Exposure to second-hand smoke contributes to lung cancer and coronary heart disease in non-smoking adults, sudden infant death syndrome, and respiratory illnesses across the lifespan; indeed, any level of exposure to second hand smoke (SHS) is a risk for disease [1]. The way to reduce SHS exposure is to limit smoking in public and private spaces via smoke-free policies.

A smoke-free policy can be defined by Article 8 of the World Health Organization Framework Convention on Tobacco Control (WHO FCTC) which calls for parties to adopt, implement and promote “effective legislative, executive, administrative and/or other measures, providing for protection from exposure to tobacco smoke in indoor workplaces, public transport, indoor public places and, as appropriate, other public places” [2]. Such measures can be defined as guidelines that reflect legislated or regulated policies, as well as voluntary, self-regulated policies to require spaces to be smoke-free [3]. Smoke-free policies can be categorized as those that impact public areas and those that impact private areas [4]. Internationally, there has been an increase in the introduction of smoke-free policies [5,6], such as bans on: smoking in enclosed public places such as public transit, office buildings, cars, shopping centers, and schools; and certain outdoor public places. Smoke-free policies may range from total bans that prohibit smoking in all indoor and outdoor areas under policy jurisdiction, to partial bans such as those with designated smoking areas or in certain service settings. Previous studies have shown that total smoking bans are more effective for smoke free compliance than partial bans [7,8].

Modern smoke free laws first came into practice during the 1990s [3]. Today, 48 countries have comprehensive policies in place [4]. High income countries have been leaders in developing and adopting comprehensive smoke-free laws with 70% having tobacco control policies including smoke-free laws. Low and middle-income countries generally have less comprehensive legislation in place [4] and therefore less evidence of enforcement strategies. When introduced, the enforcement of smoke-free policies is integral to compliance with the policies; however, the rate of compliance drops over time. 

Smoke-free policies are supported by strong evidence of measurable benefit on health outcomes. They are effective in decreasing exposure to second-hand smoke, resulting in improvements in air quality and reductions in negative respiratory symptoms [9]. Although smoke-free policies have been shown to have public support and benefit health, enforcing smoke-free settings has its challenges. As part of its policy recommendations, the WHO lists the following as examples of activities required for implementation and enforcement of smoke-free environments: information packages clearly outlining a business owner’s responsibilities under the law, including signs required under the law; designated inspectors; a “grace period” during which violators are warned and given the opportunity to comply before formal enforcement actions are taken; a procedure for the public to report violations (e.g., toll-free telephone line); and enforcement of the law should be communicated to the public [10].

Enforcement strategies are any activity designed to aid compliance with the smoke-free policy. Compliance can be measured as a reduction of smoking in the designated smoke-free spaces (i.e., people abiding by the policy) [11]. Through increasing compliance, enforcement strategies can also aid in overall implementation of smoke-free policies. Circumstances other than enforcement strategies that may impact compliance with policy are awareness of the policy, number of smoking management/teams, leadership and coordination. Enforcement strategies can range from those that do not attempt to seek out, monitor, or cite violators (e.g., signage, removing receptacles, educational campaigns and promotion of policy) to those that actively attempt to seek out, monitor and cite violators (e.g., compliance checks, issue of warnings or citations, designated complaints process, and fines), and are considered important to the initial success and adoption of a smoke-free policy [12]. However, there is no comprehensive review of the literature guiding adoption and use of effective enforcement strategies, or measures to improve policy compliance. 

### Aims

This paper aims to systematically review:

1. the effectiveness of enforcement strategies at increasing compliance with and implementation of smoke-free policies;
2. the circumstances associated with compliance with smoke-free policies (other than enforcement strategies).

## 2. Methods

The review followed PRISMA standards for the design and reporting of systematic reviews. A protocol for this review was registered with PROSPERO International Prospective Register of Systematic Reviews (Identifier: CRD42016016090).

### 2.1. Search Strategy

Medline, Medline in Process, The Cochrane Library, Embase, PsychInfo, and CINAHL databases were searched for relevant studies published between January 1980 and July 2017. MeSH terms (smoke-free policy OR tobacco smoke pollution) were combined with the following keywords using the AND command (adherence OR compliance OR implementation OR evaluation OR strategy OR environment OR second-hand smoke OR public health OR organizational policy). Previous reviews of relevant literature, the grey literature databases “Greynet” and “OpenSIGLE”, and Google Scholar (first 100 listings) were also searched along with a manual search of the reference lists of retrieved articles.

### 2.2. Definitions

The terms enforcement, implementation and compliance are interchangeable and often defined as the same activity. For this paper, enforcement strategies were defined as any activity designed to aid compliance with the smoke-free policy. Compliance can be measured as a reduction of smoking in the designated smoke-free spaces (i.e., people abiding by the policy) [11]. Implementation is the activity involved in introducing a smoke-free policy. An important distinguishing feature is that smoke free policies are implemented and enforced by the service or staff at which they are based, whereas compliance with the policy usually refers to smokers’ adherence to the rule to not smoke at a specific location. It is common for smoke free policies to be implemented, with no enforcement strategies put into place. This review is examining papers that report smoke free policy implementation and the enforcement strategies used to increase smoker compliance with the policy.

### 2.3. Inclusion Criteria

To meet inclusion, studies were required to assess the effectiveness of strategies used to enforce smoke-free policies or circumstances that impact on policy compliance. Studies in all types of environments or organizations where policies may be implemented were considered. The review was restricted to quantitative studies (including observational, cross-sectional, cohort and intervention) published in English.

### 2.4. Data Extraction

The titles and abstracts of all identified papers were assessed for relevance independently by two reviewers (first search: author A.G. and A.M.J.D.; second search: O.W. and A.M.J.D.) and rejected on initial screening if the study did not appear to meet the inclusion criteria. Studies meeting the inclusion criteria were subject to a full text review independently by two reviewers (first search: author A.G. and A.M.J.D.; second search: O.W. and A.M.J.D.), and discrepancies or uncertainties were resolved through consultation with a second author (L.T.). One reviewer (first search: A.G.; second search O.W.) also searched the reference lists of studies identified for inclusion in the review. Data from included journal articles were extracted into summary tables by one reviewer (first search: AG; second search O.W.) and a random 25% checked by a second (L.T.). Data extracted from the articles included: authors, publication year, study location, setting, sample description, methods, policy description, policy enforcement findings and policy compliance findings.

### 2.5. Methodological Quality Assessment

The methodological quality of studies was assessed using the Effective Public Health Practice Project Quality Assessment Tool for quantitative studies [13]. Two reviewers assessed study quality and any disagreement was resolved through discussion. This tool is recommended for use with public health, health promotion, and prevention research, and although it has limitations when used with studies describing behavioral outcomes or population-level interventions (e.g., inability to blind, limited validity of self-report), it is the most appropriate tool available [14]. Studies are rated as “weak”, “moderate”, or “strong” against six components: selection bias (sample representativeness and consent rate); study design; control of confounders; blinding (whether assessors were blind to participant condition and whether participants were blind to the research questions); data collection methods (whether data collection tools used were shown to be valid and reliable), and withdrawals and drop-outs (whether reasons for attrition and final follow-up numbers were reported).

### 2.6. Data Synthesis

Due to variations in study methodology and outcome measures, a narrative synthesis rather than a meta-analysis was undertaken.

## 3. Results

### 3.1. Search Results

**Figure 1:**
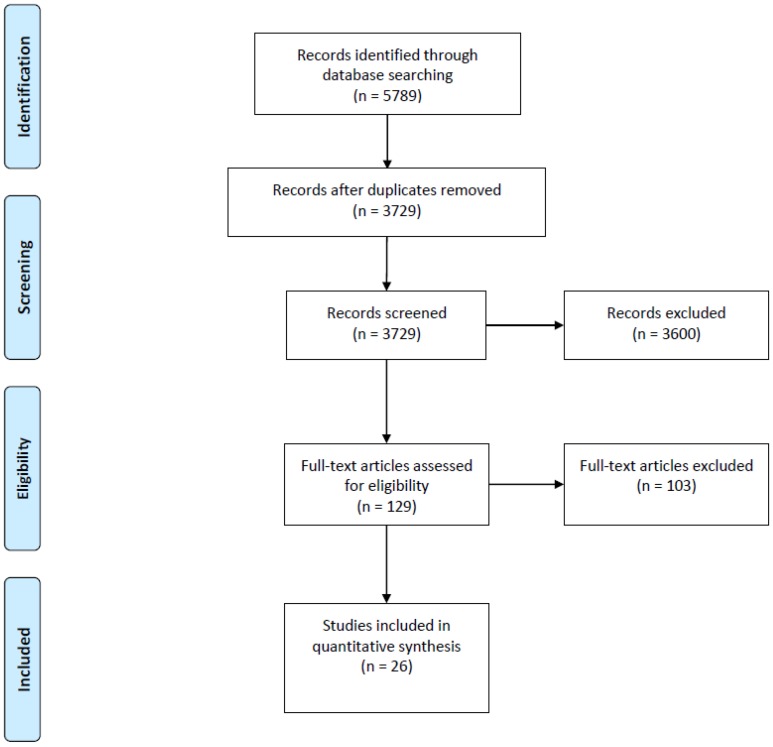
PRISMA Flow diagram.

After duplicates were removed, 3729 studies were identified from electronic searches; 2994 in the 2015 search and 735 in the 2017 search. Of those, 26 studies met inclusion criteria and were included in the review (see Figure 1). Results of the data extraction can be seen in Supplementary Table S1.

### 3.2. Study Characteristics

Of the 26 studies included, 12 were conducted in the United States, two each in Australia, China, Portugal and Spain, and one each from Canada, Greece, India, The Netherlands, New Zealand, and Thailand. Studies were based in four settings: healthcare institutions (*n* = 8, including hospitals, psychiatric services, pharmacy, and substance use disorder services); hospitality venues (*n* = 6, including studies assessing restaurants only or a combination of venues); educational institutions (*n* = 7, including schools and universities), and; public spaces and workplaces (*n* = 5, including worksites, taxis and public places). Six studies related to the behavior of the individual smoker, 19 related to the person(s) responsible for the smoke-free space, and one study included a combination of the two.

#### 3.2.1. Effectiveness of Enforcement Strategies at Increasing Compliance and Implementation of Smoke-Free Policies

*Policy promotion/policy awareness*. Of the strategies found in the papers reviewed, the strongest evidence of effectiveness was found for policy promotion/awareness. Ten studies [12,15,16,17,18,19,20,21,22,23] cited the use of policy promotion activities and/or the importance of increased policy awareness among setting staff, consumers or the general public as enforcement strategies used to increase compliance. One study used a multicomponent intervention to increase compliance with a partial smoking ban (i.e., designated smoke-free areas) on a university campus. While the intervention also involved moving cigarette receptacles, the bulk of the intervention was related to increasing awareness of the partial smoking ban by adding signs to the areas and distributing positive reinforcement cards to compliant smokers and reminder cards to non-compliant smokers. This resulted in a significant decrease in smokers in the non-smoking areas and a higher proportion of smokers moving from the non-compliant to compliant areas [15].

In two separate studies conducted in the US, mail-outs to local businesses containing information about changes to or enforcement of a smoke-free law and how to comply were used. The information mail-out interventions significantly increased awareness of smoke-free laws [18,19]. There was increased compliance observed in one study [19], and no evidence of increased compliance was observed in the other [18]. In New Zealand, the use of mass media was an important part of the enforcement strategy of associated with changes to national smoke-free legislation, with nationwide print and radio advertising (including Maori language versions), leaflets and posters, an information helpline, and a smoke-free law website used to increase awareness of the legislation which led to increased compliance. Compliance was measured by cotinine levels in customers visiting 30 bars in Auckland, Wellington and Invercargill, also by observation of smoking indoors at bars on Friday evenings before and after the legislation [12]. Self-reported data suggested that SHS exposure in the workplace decreased significantly from 20% to 8% after the new legislation. Air quality improved greatly in hospitality venues. Reported SHS exposure in homes also reduced significantly. It was concluded that the work done to promote a national smoke-free policy in New Zealand, both to the public and to key stakeholders before and during implementation assisted in high compliance [12].

On a US university campus, a targeted, theory-driven poster campaign encouraging students not to smoke in smoke-free areas was linked to a significant decrease in the number of cigarettes smoked on campus and greater campaign exposure was associated with fewer smoking violations on campus. The campaign design was guided by the theory of planned behavior with theoretical-thematic analysis being used to explore the role of theory of planned behavior constructs in effectively persuading students to comply with the smoke-free policy. There was a significant decrease in the number of observed violators during and after the poster campaign [21]. Another study conducted in a US university found a message card campaign distributed in non-compliant campus hot spots significantly increased compliance. This was defined by the number of cigarette butts counted on the ground and number of observed smokers during and post-intervention. The cards given to smokers included an efficacy enhancing messages, information on quitting resources, and coupons [22]. In another university-based study, non-compliant smokers reported significantly less policy knowledge than smokers in compliance with the policy [23]. In US high schools, teachers at schools with total smoking bans were more aware of the policy at their schools than teachers at the partial ban schools. However, there was no difference in reported smoking, therefore no observable difference in policy compliance [20].

Seven of the 10 studies citing policy promotion or policy awareness demonstrated that an enforcement strategy was implemented. Only one showed that the strategy continued to be enacted over time [12] with data collected from sources covering a four-year period. The remaining studies consisted of cross-sectional surveys [16,18,19,23], pre-post data from short-term interventions [15,21,22], and a survey after an intervention with no baseline data collected [18]. Most studies suggested that the strategy was associated with increased compliance with the relevant law or policy [12,15,17,18,21,22]; however, none achieved complete compliance. Moreover, studies of locations with total bans were more successful than those sites with partial bans (e.g., 20). Most of the studies were of weak methodological quality, with only one being considered strong [18]. Overall, the studies reviewed showed that enforcement strategies that aimed to increase policy awareness were linked to a reduction in smoking at the sites. Policy promotion is an important strategy in increasing compliance, if people and businesses are aware of their responsibilities under the law, they are more likely to comply with the law.

*Violation penalties and/or enforcement officers*. Ten studies [12,24,25,26,27,28,29,30,31,32] described the use of penalties for violations and/or the role of enforcement officers. Compliance inspections on restaurants in New York City were integrated into existing routine health department checks, and clear monetary penalties were set for violations for both owner/managers and smokers. The study assessed compliance by surveying restaurant owners/managers about their smoking policies, and physically inspecting the restaurants. Compliance was defined as the owner/manager reporting venue policies consistent with legislation, and during inspection no visible smoking or ashtrays in smoke-free areas of the restaurant. The survey found 68% of restaurants reporting compliance and the inspections found 77% compliance with smoke-free legislation [26]. In a study of public transport smoking bans, taxi drivers reported the legal ban and associated fines were the primary reasons they enforced the bans [25]. A national complaints telephone line accompanied the New Zealand Smoke-Free Environments Act [12]. Enforcement officers responded to complaints, and most complaints were resolved through letters, telephone calls and visits from enforcement staff. The study reported complaints declined steeply in the year following the roll out of the strategy package. In the US, one study reported that restaurant managers/owners were more likely to consider implementing smoke-free policies if they had received customer complaints about smoking [28].

Two studies reported on the importance of staff in policy enforcement. Smoke-free sites in the psychiatric inpatient unit setting were associated with cohesive staff enforcement, and enforcement was in turn improved by staff receiving training [27]; however, the methods of staff enforcement are not described. In the pharmacy setting, staff were responsible for policy enforcement by asking violators to stop smoking or leave the premises [24]. In Chinese hospitals, financial incentives were used for wards that were declared smoke-free, while disincentives were used when there was smoking inside hospitals, although the authors do not describe what constitutes a disincentive [29].

In the education setting, one study found that the stringency of policy enforcement was negatively related to student smoking. Levels of punishment ranged from detention to being expelled and reassigned to alternative schools [31]. One university gave campus security the authority to approach smokers, inform them of the policy, and issue monetary fines to repeat offenders [30]. Non-compliant students in one US study reported the smoke-free policy was rarely enforced and that they thought there needed to be consequences for smoking on campus [32].

Of the ten papers which included penalties and/or enforcement officers, two included enforcement officers as part of the strategy implemented in the study [12,24]. One paper collected data from 2003–2006 [12], with the others being cross-sectional surveys [24,25,26,30,31,32] or pre-post data collection after the intervention [29]. Eight of the studies found some level of compliance, with reported rates ranging from 39% [27] to 84% [25]. Five suggested that the strategy was associated with successful compliance [12,26,27,29,32]. Interestingly, two studies with similar compliance rates interpreted the results in opposition. One study, conducted in an inpatient unit found 39% compliance with smoke-free policy, and considered that to be a successful result [27]. While another, in restaurants found 40% compliance, and considered that to be low compliance [28]. All the studies were considered of weak methodological quality. Overall, the more severe the penalty, the higher the level compliance (i.e., less people smoking in smoke-free areas). Therefore, the severity of the penalty should be considered if other jurisdictions were to use penalties and/or enforcement officers as part of an enforcement strategy.

*System changes*. Less evidence was found for the effectiveness of system changes as an enforcement strategy. System change involves the change of practices and/or procedure in the workplace. Four studies [16,17,27,29] covered the use of system changes such as recording smoking status, staff education and training, and cessation support to aid policy enforcement. In psychiatric services in Spain, recording client smoking status was one of the most commonly used strategies involved in becoming smoke-free [16]. In Chinese hospitals following a similar process, the routine recording of smoking status was a strategy implemented in only a minority of the hospitals assessed [29]. In Australia, staff education and training was associated with successful implementation of a smoke-free policy and the likelihood the policy was enforced in psychiatric services [27]; however, the methods of staff enforcement are not described. Another study found low levels (<50%) of continuous staff education and training in hospitals [17], though compliance with smoke-free policy was not assessed. Another example of system changes to aid policy implementation is the use of nicotine replacement therapy (NRT) and dedicated cessation support services in the healthcare setting as part of the smoke-free policy in hospital. The studies reviewed found deficits in referrals to cessation support services and shortfalls in the use of NRT in cases where the services existed [17,27,29].

Two studies demonstrated that system change was implemented [17,29]. No studies showed that the strategy was implemented over time with three studies being cross-sectional [16,17,27] and one being a pre-post assessment of a short-term intervention [29]. Two studies showed evidence of compliance with the smoke-free policy [27,29], with those studies concluding that the strategy was associated with successful compliance to the policy. All the studies were considered of weak methodological quality. System change may be important to successful smoke-free policies; however, the evidence is limited in support of it as an enforcement strategy.

*Signage*. As part of an enforcement strategy package, six studies [16,24,25,33,34,35] reported on the use or observation of clear smoke-free signage as required by law. In the health service setting, the majority of pharmacies [24] and psychiatric services [16] reported displaying the required signage indicating those locations were in compliance with the law; however, those studies did not assess whether signage affected the compliance behaviors of individual smokers. In Portugal, required signage was independently observed by study teams in the majority of hospitality venues [34] and all taxis [25] sampled, indicating awareness of and compliance with smoke-free legislation. In India, the majority of restaurants and education settings observed did not display the required smoke-free signage, indicating low institutional compliance with smoke-free laws [33]. In Greece, the presence of signage was not found to affect SHS concentrations [35].

Smoke-free policies frequently specify that signage be displayed in areas covered by the policy. As such, most of the studies assessed the presence of signage as determined by the relevant legislation [16,26,33,34], yet only two of the studies demonstrated signage was implemented as part of an enforcement strategy [24,35]. One study assessed the use of signage over time with a two year follow up [36] while the other five were cross-sectional designs [16,24,25,33,34]. The reported smoke free compliance rates ranged from 7.2% [33] to 63% [24]. Most of the studies were of weak methodological quality. In sites with partial bans, signs may facilitate compliance as people would know the areas where smoking is prohibited. However, while the studies discussed show some level of compliance with signage requirements of smoke-free laws at the service or site, there was limited changes to the behaviors of the individual smoker.

*Planning and leadership.* Of the strategies found in the papers reviewed, the most limited evidence of effectiveness was found for planning and leadership. Two studies [17,27] considered the importance of careful planning and clear leadership and commitment in the successful implementation and enforcement of smoke-free policies. Lawn and Campion (2010) [27] found that psychiatric services that took more than six months to prepare were more likely to successfully introduce a smoke-free policy, and that clear leadership was associated with successful policy introduction. Although the paper does not define “success”, the strategies used included the provision of NRT, staff education and training related to smoking and mental illness, and enforcement of the policy by staff members. Garcia (2006) [17] reported that hospitals participating in a smoke-free project demonstrated high levels of commitment concerning all levels of leadership as required by the project protocol.

One of the two studies demonstrated the enforcement strategy was implemented [17]. Both were cross sectional and did not show the strategy continuing to be enacted over time. Both showed compliance with the strategy, with both suggesting that the strategy was associated with successful smoke-free policy. Both were considered of weak methodological quality. Therefore, evidence is limited for the role of planning and leadership in the successful enforcement of smoke-free policy.

#### 3.2.2. Circumstances Other Than Enforcement Strategies That Are Associated with Compliance

Most studies reviewed aimed to assess circumstances that may be related to compliance but are not integrated into an enforcement strategy. Some surveyed the users of the space (e.g., staff, students, patients), examining attitudes towards policies, perceptions of enforcement strategies, and/or reported strategies in use at the sites [16,22,36]. Others used pre-post assessments of enforcement strategies including surveys and observation [15,19,21,22,24,25,26,27,28,29,30,33,34,35,37]. Policy restrictiveness (total or partial ban) and extensiveness of implementation [20,24,26,32,33,34,35,36,37,38], setting type [16,25,34,35,36,39], and smoking status [18,19,25,27] were the factors most frequently identified as impacting on policy compliance. 

*Partial versus total smoke-free policy.* Nine studies [20,26,32,33,34,36,37,38,39] found that compliance with smoke-free policies was associated with whether the ban was partial or total and/or extensiveness of enforcement. Compliance was highest in settings that employed total smoking bans compared to partial bans [32,33,37,39], with only one study finding no difference [20]. One study of substance use disorder treatment organizations found that the number of required tobacco-free policies in effect (“policy extensiveness”) was related to hospital co-location, not-for-profit funding status, and level of care provided. Organizations with policies and procedures less oriented toward tobacco cessation at baseline were found to “catch-up” over time [39]. Three studies [27,33,38] reported that compliance may also be affected by how laws define smoke-free areas and subsequent changes to physical structures required to become compliant with often complicated definitions. For example, hospitality venues would only make their outdoor areas smoke-free if required to do so by law [38], and compliance was lower in restaurants that were partially enclosed compared to fully enclosed sites [33].

Of the nine studies that considered the influence of the level of ban on the success of the strategy, four compared partial smoke free bans to total smoke free bans [32,34,37,39] and all four concluded that total bans were the most effective. Most of the studies were of weak methodological quality.

*Service/venue type.* Six studies [16,27,34,35,36,39] reported that within setting types (i.e., hospitality, healthcare etc.), certain service or venue type characteristics were associated with achieving compliance with smoke-free laws and regulations. Findings were mixed in the healthcare setting. Among psychiatric and alcohol and drug treatment services, inpatient units had more extensive policies enforced than outpatient centers [16,36]. One study looking at psychiatric settings found the existence of and compliance to a smoking policy was negatively associated with employee exposure to environmental tobacco smoke and was highest among inpatient settings [39]. Compliance was assessed by a survey question asking how well others complied with the regulations. Hospitality setting venues such as bars, restaurants with bars, and nightclubs (i.e., where alcohol represented significant portion of total revenue) had lower levels of compliance than restaurants [27,34,35]. None of the studies in the current review directly compared across service type. Two studies looking at service/venue type concluded that a total ban was needed for smoke-free compliance [34,39]. The other studies did not address the issue. All the studies were of weak methodological quality.

*Smoking status.* Four studies [18,19,25,27] found compliance was higher among workplaces with non-smoking management and lower staff smoking rates. While none of the studies which mentioned smoking status of staff or management specifically addressed the effectiveness of level of ban, one study concluded that a partial ban is prone to breaches, and that clear, strong policies are needed to achieve smoke free status [25]. One was considered to be of strong methodological quality [18], one was moderate [19], with the remaining two being weak methodological quality. 

*Physical environment.* When looking at the university setting, non-compliant “hot spots” were observed to share common characteristics that appeared to facilitate smoking such as seating, areas for cigarette disposal, and reduced visibility from high traffic areas [15,30]. The two studies that considered the physical environment as effecting compliance did not discuss the potential effect of level of ban. Both studies are of weak methodological quality.

*Network membership*. Three studies [16,17,24] in the healthcare setting found that sites which were members of smoke-free networks driving enforcement of smoke-free policies were more adhered more closely with the guiding code and had more tobacco-free strategies in place. The networks were organized by non-profit organizations to support the promotion of smoke-free policies. Three studies that considered the physical environment as effecting compliance did not discuss the potential impact of level of ban. All studies are of weak methodological quality.

#### 3.2.3. Methodological Quality Assessment

Table 1 summarizes the methodological quality of the included studies. Although the tool has the capacity to provide a global study rating, the “blinding” and “confounder” criteria were not always applicable, as such robust conclusions about study quality are difficult to make. Only three out of 26 studies were rated as “strong” or “moderate” for all assessment criteria that applied [18,20,35]. While six studies utilized quasi-experimental designs [12,22,26,27,30] and some utilized multiple data sources (e.g., surveys, complaints records, and observational data) (e.g., 30), the majority were cross-sectional studies [16,24,25,27,32,33,36,38,39]. As such, study designs were largely classified as “weak”. Most studies reported using an appropriate statistical test, although studies were determined to have a unit of allocation that differed from the unit of analysis. This was generally due to all participants falling under an existing or updated smoke-free policy. Co-intervention was unlikely to have occurred in most cases.

## 4. Discussion

This review of the literature of enforcement strategies for effective compliance with and enforcement of smoke free policy found a limited and weak evidence base. No intervention studies with rigorous trial design were identified. Our review of the existing literature suggests that policy promotion and enforcement officers/penalties for violations may improve compliance with smoke-free policy in several settings. These strategies were reported more frequently in studies with both the enforcer of the policy and the smoker within the environment where a policy applied. Other strategies that may be successful but need better quality and quantity of evidence are system changes, signage, and planning. A variety of circumstances were also identified as associated with compliance with smoke-free policies. Workplaces were more likely to comply with policies where the staff were aware of the laws, had non-smoking management, lower smoking rates, and the workplaces were members of a smoke-free network. As for individual smokers, not being aware of the governing smoke-free policy, being a smoker and having access to physical structures in the environment that facilitate smoking were associated with lower levels of compliance.

The implementation of smoke-free policies holds a range of benefits including reduced levels of second-hand tobacco smoke exposure, reduced tobacco use among smokers, improved health and productivity of employees and reduced hospital admissions due to tobacco related disease [9,40]. Traditionally, there have been wholesale introductions of smoke-free policies across a range of environments and settings. Once implemented, smoke-free policies are often thought of as self-enforcing [41]. However, this is likely dependent on policy awareness which requires successful policy promotion and clear and visible signage designating a smoke-free environment as shown by the studies discussed. Several studies have outlined cases where communicating a strong and clear rationale for policy change or implementation results in widespread policy support and better uptake and compliance [42,43]. However, particularly in settings with a strong smoking culture (e.g., environments where smoking is considered more socially acceptable, such as bars and clubs) policy awareness alone may be insufficient [25,26,27,38].

The use of enforcement officers and clear penalties for non-compliance is beneficial in the successful implementation of a smoke-free policy through enforcement, as shown by studies discussed [12,27,29,30]. Often a lack of resources is cited as a significant barrier to implementation of smoke-free policy [43,44]. However, enforcement duties can be integrated into existing systems and roles, for example compliance checks conducted as part of health and safety inspections, workplace security tasked with policy enforcement, and engaging all staff in enforcement responsibilities [26,27]. Furthermore, policy implementation in the hospital setting is far more successful when all staff consistently enforced smoking bans with patients [27].

Many of the factors identified in this review as impacting on policy compliance link directly back to enforcement strategies. For example, both workplaces and individuals are more likely to be compliant if they are aware of a policy [12,16,17,18,19,36]. As for differences in compliance regarding different setting or venue types, it is often settings that have stronger smoking cultures that are less compliant. Network membership is related to the need for clear leadership and planning [16,17,24]. The finding that smoking status is associated with non-compliance [18,19,25,27] lends support to the need to consider system changes as part of policy implementation, such as offering or referring to cessation support. Confusion around how to comply with a partial ban policy also leads to non-compliance. However, the single most important factor associated with policy compliance was policy extensiveness: total bans resulted in higher compliance than partial bans. 

### 4.1. Implications

Based on the included studies it appears that successful enforcement strategies leading to increased compliance with smoke-free legislation are likely to require: sufficient time to plan comprehensive policy promotion, procurement and placement of signage; include integration of pathways for identifying and dealing with non-compliance; and consider how the policy may impact on other systems or day-to-day operations of the workplace or environment. Policy promotion may be achieved through various means such as information packs, websites, promotional days, informing people upon entry of the smoke-free policy, and most importantly clear and visible signage. At least some signage should also briefly state the penalty for non-compliance and how to report violations. Consideration should be given to the use of enforcement officers to perform compliance checks and issue warnings or penalties to violators. Enforcement can also be incorporated into other staffing roles such as security or general staff, providing staff are aware of their role and responsibilities. Penalties may include warnings, citations, monetary fines, removal from premises or other prescribed actions suitable to the setting (e.g., detention in schools). Finally, policy implementation may lend itself to other system changes within the setting. For example, the recording of smoking status and provision of cessation support in the healthcare setting, or more generally the provision of staff education and training around the policy and support for staff to quit in addition to other strategies including signage and awareness-raising. Such strategies should be tested in appropriately designed trials such as cluster randomized trials, stepped wedge designs or interrupted time series to strengthen the evidence base.

### 4.2. Limitations

Due to the variability in study designs and outcome measures, a meta-analysis of the results of studies could not be conducted limiting the review to a narrative synthesis. Additionally, although the methodological rating tool used does provide a global study rating, we were unable to employ this due to the study designs rendering some rating categories not applicable. The tool is recommended for use with public health, health promotion, and prevention research; however, it has limitations when used with studies describing behavioral outcomes or population-level interventions, such as inability to blind, limited validity of self-report. Nevertheless, it is considered to be the most appropriate tool available [14]. Another limitation of the current review is that qualitative studies were not included in the current review. Qualitative studies allow for the investigation participants’ individual experience and interpretation of the intervention or environment, thereby producing complex data with context [45]. Future reviews could expand the scope to include qualitative studies to capture the participant experience of smoke-free policies.

## 5. Conclusions

This review provides a summary of the use of enforcement strategies for smoke-free policies as well as circumstances that may impact on compliance. The most frequently cited enforcement strategies for the successful implementation of smoke-free policies were policy promotion and awareness-raising activities, clear and visible smoke-free signage, and the use of enforcement officers and/or penalties for violations. In terms of compliance, workplaces were more likely to comply with smoke-free policies where they were aware of the laws, had non-smoking management, lower staff smoking rates, and were members of a network guiding the policy implementation. Overall, partial bans were less effective than total smoke-free bans, as total smoke-free bans were more likely to be complied with compared to policies that had only partial restrictions.

## Figures and Tables

**Table 1:** Assessment of methodological quality.

Study	Selection Bias	Study Design	Confounders	Blinding	Data Collection	Withdrawals
Balbe et al., 2012 [16]	M	W	*	*	S	N/A
Boris et al., 2009 [20]	M	M	S	M	S	N/A
Eby et al., 2013 [36]	W	W	*	*	S	N/A
Edwards et al., 2008 [12]	W	W	*	*	M	N/A
Fallin et al., 2013 [22]	W	M	*	M	S	N/A
Garcia et al., 2006 [17]	M	W	*	*	S	N/A
Harris et al., 2009 [15]	M	W	*	*	M	N/A
Hyland et al., 1999 [26]	M	W	*	*	S	N/A
Jancey et al., 2014 [30]	M	W	*	*	S	N/A
Kaur et al., 2014 [33]	M	W	*	M	S	N/A
Kennedy et al., 2009 [38]	M	W	*	*	S	N/A
Lawn et al., 2010 [27]	M	W	*	*	S	N/A
Nimpitakpong et al., 2010 [24]	W	W	*	*	W	N/A
Paek et al., 2013 [31]	M	W	*	*	S	N/A
Record et al., 2017 [21]	W	M	*	W	M	M
Ravara et al., 2013 [25]	M	W	*	*	W	N/A
Reis et al., 2014 [34]	M	W	*	*	W	N/A
Rigotti et al., 1992 [18]	S	S	S	M	S	N/A
Rigotti et al., 1994 [19]	M	M	S	W	W	N/A
Russette et al., 2014 [23]	M	W	*	*	S	N/A
Sorensen et al., 1992 [32]	M	W	*	*	M	N/A
Stillman et al., 2013 [37]	M	M	W	W	S	N/A
Vardavas et al., 2013 [35]	M	M	S	M	S	M
Willemsen et al., 2004 [39]	W	W	*	*	S	N/A
Williams et al., 2004 [28]	W	W	*	*	M	N/A
Xiao et al., 2013 [29]	M	M	W	W	W	N/A

Note: W (weak), M (moderate), S (strong) and * (absent).

## Supplementary Materials

The following are available online at http://www.mdpi.com/1660-4601/15/7/1386/s1, Supplementary Table S1: Data extraction.
