# Supplementary material for: Signs, Fines and Compliance Officers: A Systematic Review of Strategies for Enforcing Smoke-Free Policy
Source: Int J Environ Res Public Health. 2018 Jul 2;15(7):1386. doi: 10.3390/ijerph15071386 (PMC6068603; doi:10.3390/ijerph15071386)
Supplement: Supplementary file 1 [file ijerph-15-01386-s001.pdf]

Table S1. Data extraction.

**Authors (Year) Study Location**	**Setting**	**Sample Description** (A) Individual (B) Responsible party	**Methods**	**Policy Description**	**Policy Enforcement**	**Policy Compliance**
Ballbe, M., et al. (2012)<br><br>Catalonia, Spain	Psychiatric services (inpatient units and day centres)	**(B)** Clinical managers	Cross-sectional survey of public psychiatric service centres in Catalonia, Spain. Managers (N = 186) were surveyed by a 24 item self-report questionnaire covering four dimensions - clinical intervention, staff training and commitment, smoking area management and communication of smoke-free policies. There was a 96.9% response rate.	Conducted in context of Spanish law extending existing ban (smoking banned in indoor public places and workplaces, including hospitals) to outdoor hospital campuses and also smoking areas (either indoor or outdoor) in short-stay psychiatric units.	**Most commonly implemented strategies** (≥75% sample 'always/often'):<br>- Smoking recorded on medical file<br>- Managers promoted awareness-raising strategies targeting staff<br>- Smoking prohibited in common indoor areas<br>- Clear signage indicating smoking/no-smoking areas<br>- Changes in smoke-free policies communicated to staff and patients<br>- Patients informed of benefits of smoke-free policies	**Membership to smoke-free network increased enforcement of policies:** Membership to a network of Smoke-free Hospitals requiring members to commit to a progressive implementation of tobacco control strategies significantly increased probability of hospitals providing smoking intervention to staff and patients, providing staff training and communication of smoke-free policies.<br><br>**Service type associated with enforcement of smoke-free policies:** Day centres that saw patients daily over long periods had lowest amount of smoking intervention strategies, staff training and commitment and communication of policies compared to inpatient units.
Boris, N., et al. (2009)<br><br>Louisiana, US	Schools (with different smoking policies)	**(B)** Teachers and students	Case study (surveys) using data from the baseline survey of The Acadiana Coalition of Teens Against Tobacco (ACTT) study (Johnson, Myers, Webber, & Boris, 2004). Data from teachers (n = 1,041) and ninth-grade students* (n = 4,763) at 20 schools in five districts in Louisiana. The survey included demographic information, tobacco use history, knowledge and attitudes about their school's smoking policy. Response rate was not reported.<br>**only smoking status assessed*	Comparison of 'restricted-use' and 'no-use' policies.<br>**Restricted-use:** allow teachers and other staff to smoke in one "restricted" area on campus.<br>**No-use:** prohibition of all tobacco use by anyone on the school campus and at all school events	**Awareness of policy higher for total ban:** Teachers at no-use policy schools were more aware of the tobacco-use policy at their schools than teachers at the restricted-use schools.	**No difference** in proportion of teachers reporting smoking on campus in schools with no-use vs. restricted-use policies.

Eby, L.T.T. & Laschober, T.C. (2013)<br><br>New York, US	Substance Use Disorder treatment organisations	**(B)**<br>Clinicians	Repeated cross-sectional surveys measured substance use disorder clinicians' perceptions regarding the implementation extensiveness of the Office of Alcohol and Substance Abuse Services (OASAS) tobacco-free regulation, passed in New York State in July of 2008. Repeated cross-sectional data were collected at approximately 4 months pre-regulation (time 0, n = 362, 74% response rate), 10–12 months post-regulation (time 1, n = 462, 74% response rate), and 20–24 months post-regulation (time 2, n = 509, 83% response rate).	Tobacco-free regulation requires all programs to be 100% tobacco-free. Applies to patients, visitors, volunteers, and employees and prohibits tobacco in indoor facilities, outdoor grounds, and vehicles owned, operated, or leaded by the organisation. Also prohibits any individual from brining tobacco products into the treatment program, and the treatment organisations are required to offer tobacco cessation treatment.	N/A	**Hospital-based status, profit status, and level of care offered associated with perceived implementation extensiveness (number of required policies in effect), tobacco-related intake procedures and cessation counselling; although patterns differed across the 24-month period.** Organisations with policies and practices that are less oriented toward tobacco cessation prior to the tobacco-free regulation began to "catch-up" over time.
Edwards, R., et al.<br>(2008)<br><br>New Zealand	National smoke-free policy relating to indoor workplaces and public places, excluding schools and early learning centres	**(B)**<br>Mixed data sources	Multi-faceted evaluation conducted by extracting, appraising and summarising evidence from mixed data sources including monitoring surveys; stakeholder surveys; indoor air quality study; hospitalisation rate data; Quitline data; econometric data.	The New Zealand 2003 Smoke-free Environments Amendment Act extended existing restrictions on smoking in office and retail workplaces by introducing smoking bans in bars, casinos, members' clubs, restaurants and nearly all other workplaces.	**Process of monitoring and enforcement was largely passive.** Enforcement officers working within district health boards investigated complaints received largely from members of the public either directly to district health boards or through a national complaints telephone line. The Ministry of Health was responsible for prosecuting persistent offenders. There was no pro-active monitoring and inspection schedule.<br><br>**Most complaints resolved through letters, telephone calls and visits from enforcement staff.** Complaints went from 75 in first month post regulation, to fewer than 20 per month one year later.<br><br>**Awareness campaigns used to inform public of changes to legislation.** Prior to implementation mass media campaigns were run on television supported by print and radio advertising, leaflets and posters,	**Authors suggest compliance was helped by:**<br>- Broad support for legislation<br>- Work to promote and explain the policy to the public, and involve and inform key stakeholders that started well before and continued during implementation

					an information helpline, and a smoke-free law website, as well as by additional promotional material produced by non-governmental organisations.	
Fallin, A. (2013)<br><br>Kentucky, US	University	**(A)**<br>312 observational periods at 39 campuses	Pre-post quasi-experimental design using purposive sampling. Compliance was operationally defined as<br>the number of cigarette butts counted on the ground and number of observed smokers<br>Policy compliance was measured using the Tobacco-Free Compliance Assessment Tool. Field notes were used to assess campaign reaction.	Tobacco-free campus policy: prohibiting the use of all tobacco products on all university property.	N/A	**Message card campaign used to increase compliance:** Cards containing efficacy-enhancing messages with a web link to quit smoking resources were distributed at campus hotspots for non-compliance. Compliance (measured by number of cigarette butts) significantly increased during and post-intervention.
Garcia, M. (2006)<br><br>Catalan, Spain	Hospital	**(B)**<br>Hospitals in the implementatio n or consolidation stage of network membership to the Catalan Smoke-free Hospitals Project	Self-audit questionnaire – "Self-Audit Questionnaire of the European Network for Smoke-free Hospitals". Each hospital (*n* = 25) was analysed according to the duration of its Network membership (< 1 year: implementation stage; Z1 year: consolidation stage). Response rate is not reported.	Smoking in hospitals is prohibited under both national and regional legislation.<br>The Catalan Network of Smoke-free Hospitals follows the European Network for Smoke-free hospitals 'European Code and Standards for Smoke-free Hospitals'. The code provides hospitals with clearly defined standards and a supportive instrument to assist in going smoke-free and covers commitment concerning all levels of leadership, communication of the project requirements internally and externally, introduction of tobacco control measures step-by-step, baseline and follow-u assessments and ensuring quality assurance.	Hospitals participating in the Smoke-free Hospitals Project demonstrated high levels of implementation on the following code standards:<br>- Commitment<br>- Understanding among staff, patients and visitors that the hospital is a smoke-free organisation<br>- Compliance with no-smoking regulations in working areas, cafeterias and other communal areas used by staff, patients and visitors as well as with the separation between designated smoking and no-smoking areas<br>- Widespread implementation of no-smoking environments<br>Indicators that had levels of implementation below 50%:<br>- Continuous staff education and training with respect to smoking<br>- Health promotion: the involvement of hospitals in antismoking activities at the local, national and international level, and the promotion of smoke-free activities outside the organisation	**Network membership:** hospitals who had been a member of the Catalan Network of Smoke-free Hospitals for ≥1 years (i.e. consolidation phase of smoke-free hospitals project) had significantly higher levels of implementation of:<br>- Informing and communicating to patients, visitors and health professionals that the hospital is smoke-free<br>- Presences of smoke-free signs, clear identification of smoke-free areas and reducing exposure to SHS<br>- Done more to establish health workplaces<br>- Developed mechanisms for evaluating and monitoring the project

					- Health workplaces: i.e. new staff being informed during recruitment that hospital is smoke-free; whether smoke-free obligations have been included in existing disciplinary procedures.	
Harris, K. J. et al (2010)<br><br>Montana, USA	University campus	**(A)** people on campus – not specified whether students, staff, or visitors (n= 709)	Pre-post assessment of an enforcement package by observing the number of complaint and non-compliant smokers before, during, and after the strategy package was in place	partial ban – within 25 feet of a building	Individual smoker compliance – multiple component intervention<br>- moving cigarette receptacles outside the 25-foot smoke-free zone<br>- specifying the smoke-free zone with prominent ground markings,<br>- adding signs about the outdoor smoking ban, and<br>- Distributing positive reinforcement cards to compliant smokers and reminder cards to noncompliant smokers. Positive reinforcement cards thanked smokers for their compliance and were redeemable for a free beverage at the student union building.	The proportion of smokers who always complied with the outdoor smoking ban was 33% during baseline, increased to 74% during intervention, and was maintained at 54% during follow-up.<br><br>Overall there was a strong, statistically significant association between intervention period and compliance proportions ($p <.001$)<br><br>There was a higher proportion of smokers moving from the noncompliant to compliant areas (7.2%), compared to baseline or follow-up weeks (2.3% and 2.6%, respectively).
Hyland, A., Cummings, K.M., & Wilson, M.P. (1999)<br><br>New York, US	Restaurants	**(B)**<br>Mixed data sources	Multi-faceted evaluation consisting of telephone survey with restaurant owner/managers (n=251) and inspections of the 251 surveyed restaurants, and Department of Health complaint records. Response rate of 60%.	New York City's Smoke-Free Air Act (1995): Regarding restaurants – smoking is prohibited in the indoor dining area of restaurants with >35 indoor dining seats. Those with ≤35 indoor dining seats and stand-alone bars or taverns where alcohol is ≥40$ of total revenue are exempt. Smoking is permitted in the bar areas of restaurants; however, there must be at least a six feet gap between bar and dining areas or a ceiling-to-floor partition/wall between the areas. Other specifications about square footage of smoking spaces, smoking rooms and outdoor seating areas also apply.	Restaurant owners/managers responsible for enforcing the law, and the New York City Department of Health performs compliance inspections as part of the routine health department check administered to all licensed restaurants. Penalties for violation range from $200 - $1000 for owner/managers, and $100 for smokers.	**Restaurants with bars were significantly less likely to be fully compliant.** Unclear if this was due to owner/manager active violation of law, or confusion over specific aspects of the law.<br><br>**Actions taken to become compliant:**<br>- Posting signs where smoking is and is not permitted (82%), starting to serve food in the bar area (26%), installing air filters (21), and placing a cigarette butt receptacle outside (20%).
Jancey, J., et al. (2014)	University	**(A)**<br>Mixed data source	Multi-faceted evaluation consisting of an environmental audit of	Total smoke-free policy encompassing the entire university grounds of the main	Prior to and during implementation:<br>- A University website providing information on campus cessation	**Smoking 'hot spots' where non-compliance was observed shared the following characteristics:** physical

Perth, Australia			one campus; direct observations of smoking; and intercept interviews with smokers (n=37 students & staff) conducted over a five day period.	campus, including student housing.	courses and link to off-campus quit programs<br>- Banners placed around campus<br>Added during implementation:<br>- Distribution of flyers and posters<br>- Advertising in university magazines, newsletters, and handbook<br>- University promotional days<br><br>**Security patrol the campus and approach smokers to inform them of the by-laws. They have the authority to issue warnings and fines to repeat offenders of up to $100 per infringement.**	structures for seating or leaning against, areas to dispose of the cigarettes, reduced visibility, 'isolated' from main campus area, close to student computer labs.<br><br>**Non-compliant smokers surveyed were:** male (84%), born outside of Australia (76%), international students (52%) and all were aware of the smoke-free policy.
Kaur, P., et al. (2014)<br><br>Chennai, India	Restaurants<br><br>(Educational institutions – to lesser extent)	**(B)**<br>Restaurants and educational institutions	Cross-sectional survey of Restaurants (n=400) and educational institutions (schools and colleges; n=287) using observation checklist.	Cigarettes and Other Tobacco Product Act (2003): included a ban on smoking in public places and on sale of tobacco around educational institutions.	Display of signage required under law – majority of restaurants (93%), schools (97%) and colleges (85%) observed did not display signage.	Authors observed (no statistical tests of significance) smoking was more common and signage compliance lower in partially enclosed restaurants as compared to enclosed restaurants (even though ban applies equally)
Kennedy, R.D, et al.<br>(2010)<br><br>Ontario, Canada	Restaurants & Bars	**(B)**<br>Hospitality sector operators/owners (n=403)	Cross-sectional survey conducted via telephone of operators/owners (72% response rate) followed by 20% of venues randomly selected for a visit in person to verify smoking status and physical environment.	The Smoke-Free Ontario Act (206) included restrictions that banned smoking in outdoor public places or workplaces with roofs, overhangs, or awnings, but still permitted smoking if such structures were not present. Venues that had permitted smoking and had a roof were required to either go 100% smoke-free or to alter their space physically to comply with the act. The maximum corporate fine listed in the Act for non-compliance is up to $300,000.	N/A	**Majority of hospitality venues would only make their patios smoke-free if they were required to do so by law.**<br>There was a significant increase in the number of smoke-free patios following implementation of the law. Increase in the proportion of smoke-free patios were significant for venues classified as 'restaurant and bar', 'restaurant only' and 'family restaurants'. There was no change for the bars in the sample.<br><br>For existing venues with physical structures, half made physical changes to their environment to become compliant, while the other half went smoke-free (51% of these became smoke-free due to cost of changing their outdoor environment).
Lawn, S. & Campion, J. (2010)<br><br>Australia	Psychiatric Inpatient Units	**(B)**<br>Clinical staff with responsibility for 99 adult psychiatric inpatient units (56 open units;	Semi-structured in-depth interviews with 60 administrators conducted via telephone between Oct 2007 and July 2008.	The sample consisted of open and locked units in 4 of 15 (26%) Australian stand-along psychiatric hospitals, 63 of 109 (58%) speciality adult psychiatric units within general hospitals, 2 of 6 (33%) Australian veterans' hospitals, and 3 drug and alcohol detoxification inpatient units.	**Planning**: Sites that took **more than six months to prepare were more likely to successfully implement a smoke-free policy** than those taking less than six months.<br><br>**NRT**: sites offering combinations of NRT products to individual patients were more	Association between staff smoking rates and failure of smoke-free initiatives. Reduced rates of staff smoking were associated with better leadership, more extensive staff education and training about mental illness and smoking, and more cohesive teamwork.

		43 locked units)		Sites were categorised according to whether they had successfully gone smoke-free (39%), were actively planning to become smoke-free (15%), had attempted to go smoke-free and failed (14%) or were not currently planning to be smoke-free (31%).	likely than sites that did not to be smoke-free.<br><br>Factors associated with successful implementation of a smoke-free policy: **clear leadership, cohesive teamwork, staff education and training related to smoking and mental illness, and enforcement of the policy by staff members.**<br><br>**Level of staff education and training about smoking and mental illness was associated with whether they enforced smoke-free policy.** (Units with training more likely to enforce the policy)	Whether NRT was offered to staff was associated with smoke-free status.<br><br>Consistency in which staff enforced smoke-free policy was associated with successful implementation. (For units that reported failed implementation, none reported consistent policy enforcement)
Nimpitakpong, P., et al. (2010)<br><br>Thailand	Drugstores	**(B)**<br>Drugstores	Cross-sectional survey which was self-administered via mail (n=1001). Surveys were sent to 3600 drugstores (n=1700 that participated in the Thai Pharmacist Network for Tobacco Control; n=1900 that did not) 27.8% response rate. Survey questions related to - smoking prevalence on the site, display of no-smoking signs, and the number of stores selling cigarettes.	Non-Smoker Health Protection Act (1992): ban on smoking inside enclosed public places and building operating an air-conditioning system. Also required no-smoking signs to be displayed at the entrance and inside the building where appropriate. (However, more than half of drugstores in the country were exempt as they were not air-conditioned).<br><br>In 2006, legislation that required all drugstores (air-conditioned or not) to be smoke-free was passed.<br><br>The Thai Pharmacy Network for Tobacco Control launched a 100% smoke-free drugstore campaign to introduce the 2006 regulation through the dissemination of necessary materials (e.g. no-smoking signs). The campaigns' application forms, pamphlets and no-smoking signs were distributed to pharmacy owners and staff at professional meetings, conferences, and through the governmental body at the time of licensing renewal.	Signage: most signs were posted at the entrance door or hallways<br><br>Enforcement: Staff asked smokers to stop smoking or leave the store.	**Network membership:** Drugstores participating in the smoke-free campaign run by the Thai Pharmacy Network for Tobacco Control possessed smoking signs, displayed smoking signs and their staff enforced the policy significantly more often that non-participating drugstores.

Paek, H-J., Hove, T., & Oh, H.J. (2013)<br><br>Michigan, US	Schools	**(A)**<br>Students	Multi-level analysis merging: individual-level data from the 2009 Michigan Youth Risk Behavior Survey, and school-level data from the 2008 School Health Profiles survey. The merged data resulted in *n*=1088 students nested within 14 high schools.	The Tobacco Section of the Michigan Department of Community Health strategic plan cites the implementation of "24/7" tobacco-free school policy in all Michigan schools. A 24/7 TFSP prohibits the use of any tobacco products at all times on school property, including school vehicles, and at all on- and off-campus school sponsored athletic and extramural events.	**Stringency of tobacco policy enforcement** negatively related to individual students' smoking: the higher the level of punishment, the less likely individuals students smoke. Enforcement strategies included:<br>- Placed in detention<br>- Not allowed in extracurricular activities<br>- Given in-school suspension<br>- Suspended from school<br>- Expelled from school<br>- Reassigned to alternative school<br><br>**Antismoking communications** negatively related to individual students' smoking.	A **high level of smoking** at schools was associated with individual students' smoking.
Ravara, S.B., et al. (2013)<br><br>Lisbon, Portugal	Taxis (motor vehicles/public transport)	**(B)**<br>Taxi drivers	Cross-sectional study with purposive sampling consisting of structured interviews and direct observation while using taxi services (n=250). 98.8% participation rate.	In 2008, Portugal implemented a partial smoking ban. Smoking areas are allowed in hospitality venues and shopping malls and many exemptions are accepted. Smoking is banned in workplaces and public transport.	All taxis displayed the required **signs** concerning the legal ban.<br><br>**Introduction of ban resulted in significant reduction of drivers allowing smoking in their taxi** (77% prior ban vs. 17% post ban, p<.001).<br><br>**Legal ban and associated fines** was the primary reason for drivers not allowing smoking in their taxi.	**Smoking status** of taxi driver associated with self-enforcement and compliance. Smokers significantly more like to smoke in the taxi and to allow passengers to smoke.<br><br>Heavy smoking, working night-shift and allowing smoking in taxi prior to the ban predicted non-compliance post-ban implementation.
Record, R. A. et al (2017)<br><br>Kentucky, USA	University campus	**(A)** students	Quasi-experimental, time-series design using survey measures and observational counts with assessment at three weeks pre-intervention, four weeks during the intervention, and three weeks post-intervention. Response rate for the pre-intervention survey was 10.6%. The response rate for the post-intervention survey was 59%. 284 students completed both surveys.	no specific description of the policy beyond "a University's tobacco-free policy".	Individual smoker compliance – posters and yard signs around campus designed using Theory of planned behaviour	Individual level<br>- Significant decrease in the number of cigarettes smoked on campus (p < .001)<br>- greater campaign exposure associated with fewer smoking violations on campus (p < .01) population level<br>- significant decrease in the number of observed violators pre-intervention and during the intervention (p < .001) and the number of observed violators pre-intervention and post-intervention (p = .001).
Reis, M.F. (2014)<br><br>Portugal	Hospitality venues	**(B)**<br>Observations of hospitality venues	Observational cross-sectional study of a random sample of venues (n=1394) assessing	Portuguese smoke-free law (2008) targets all indoor public places and workplaces. A partial ban applies to leisure-hospitality	Majority of venues adopted a total ban policy (76%), 16% opted for smoking permission, and 8.4% had designated smoking areas.	**Strength of ban:** noncompliance was higher in the venues where smoking was fully permitted (34%) and was lower in

	(cafeteria/pastry, restaurant, nightclub/bar)		indicators of noncompliance with Portuguese smoke-free law (e.g. lack of signage, ashtrays, people smoking, cigarette butts).	sector: public venues smaller than 100m² can allow smoking provided ventilation and exhaust systems are in place, while larger venues are compulsorily smoke-free but can adopt designated smoking areas that do not exceed 30% of the total area.	**Signage** was visible in majority of venues observed.	those venues that adopted a total ban policy (8%).<br><br>**Type of venue:** discos/bars/pubs showed highest noncompliance, especially in venues with a designated smoking area. Restaurants had a lowest noncompliance when total ban was adopted.
Rigotti, N.A., et al.<br>(1992)<br><br>Brookline, US	Workplace	**(B)**<br>Businesses with ≥5 employees (i.e. falling under smoke-free law) randomised (n=535). Surveys completed with n=345 eligible businesses.	RCT of mail-out to town businesses. 535 eligible businesses were randomly assigned to one of three groups – control (no mail out), two intervention groups (both sent information about the bylaw, one group asked to return a copy of their written policy). A telephone survey of awareness and attitudes to the law was conducted three months after the mail out. The response rate for the survey was 87%.	Town bylaw (1988) restricted or banned smoking in work sties and public places and enlarged existing restaurant non-smoking sections from 25% to 50% of seats. The bylaws applied to businesses with ≥3 employees. The law required employers to establish and post a worksite smoking policy, they were allowed but not required to designate smoking areas so long as they did not expose non-smoking employees or the public to smoke, and no-smoking signs were required. Implementation and enforcement were the responsibility of the health department.	**Mail out**: The packet sent to businesses in the intervention groups included (1) a copy of the bylaw, (2) a cover letter from the health director summarizing what the bylaw required of employers, and (3) a sample smoking policy. One intervention group additionally received a request by the health commissioner to return a written copy of its smoking policy (mailing + surveillance).<br><br>Companies sent the mail out (compared to controls) were more likely to:<br>- Know about the law<br>- Cite the law as a reason for their businesses smoke-free policy adoption<br><br>**Mail out intervention increased awareness of smoke-free law.**	**Awareness:** businesses aware of the law were significantly more likely to comply with it.<br><br>By itself, the mailing did not clearly increase compliance with the law, and the effect of the mailing was not enhanced by surveillance.<br><br>Smoking rules did not differ significantly between companies sent the mailing and controls.<br><br>Companies sent the mailing were more likely than controls to self-report full compliance and a written smoking policy, however this was not support by direct observation data.<br><br>Restrictions were more common in:<br>- Service businesses<br>- Firms whose top manager did not smoke<br>Bans were more common in:<br>- Companies with a non-smoking manager<br>- Companies with fewer smoking employees
Rigotti, N.A., Stoto, M.A., & Schelling, T.C. (1994)<br><br>Cambridge, US	Workplace	**(B)**<br>Two independent samples of businesses with ≥1 employee and not located in the home falling under the ordinance of restricted	Telephone surveys of two independent samples (1) 3-months and (2) 24-months after smoke-free law went into effect. (3-month survey: n=312; 24-month survey: n=317). Owners/managers completed the surveys which consisted of questions about	No-smoking ordinance for Cambridge, Massachusetts: required employers to ban smoking in any room "which employees normally frequent during the course of employment, including, but not limited to, work areas, employee lounges and restrooms, conference rooms, and hallways." Employers may, but are not required to, provide an	Law was considered to be self-enforcing. Only implementation activity: to inform businesses, the health commissioner used **news media and mailed a fact sheet** to the members of the chamber of commerce, about half the businesses in the city.<br><br>Awareness of the law declined over the 2 years (92% vs 73%), as did knowledge of its general provisions (64% vs 49%).	At 2 years after implementation, compliance was better in companies with a **non-smoking top manager** and a non-smoking respondent. Businesses that knew of **(awareness)** and favoured the law were also more likely to comply with it.

		smoking in worksites	awareness and opinion about the law, their perception of the law's effect on employees, air quality, and cost.	area for smokers, so long as it does not expose non-smoking employees or the public to smoke. No smoking signs must be conspicuously displayed. Employers who knowingly violate the law can be fined ($25-100) or lose any city license.	Awareness of the law 3 months after adoption was independently associated with only one factor:<br>- Companies that recalled the city's fact sheet were better informed than those that did not.<br>At 2 years, larger business and non-smoking respondents were more likely to know about the law.	
Russette, H.C., et al.<br>(2014)<br><br>Missoula, US	University	**(A)**<br>Students and non-students	Intercept interviews using a 22-item measure with closed- and open ended questions conducted with smokers (n=60) observed smoking in both compliant and non-compliant areas at one university with a 100% tobacco ban.	University policy banning all tobacco use on campus and in all campus-owned facilities.<br>- 2-year planning period prior to implementation involved: a campus-based advisory committee explored possible policies, assessed support for a new policy, hosted student debates, released publicity about new policy consideration, and drafted the potential policy for campus-wide discussions.<br>- Policy became active 1-year after it was enacted. During lead up to policy change: the student health centre led intensive media campaign promoting change and resources for smoking cessation.	Only 10% of participants reported that the policy was enforced.<br><br>In open-ended response questions regarding the factors that might influence noncompliant smokers to avoid smoking on campus, the most common response was adding **consequences** for smoking on campus property	**Policy knowledge**: the noncompliant group of smokers had significantly less knowledge about the areas where smoking was prohibited compared with the compliant smokers.<br><br>**Peer support:** significantly more compliant smokers reported that peers telling them not to smoke in prohibited areas would influence their smoking choices.<br><br>**Citations:** more noncompliant smokers reported that citations would increase their propensity to follow the rules.
Sorensen, G., et al.<br>(1992)<br><br>US	Worksites	**(A) + (B)**<br>Worksites with ≥50 employees (n=710) from 11 intervention communities participating in a larger RCT.<br><br>Employed community members (n=3143) from the 11 intervention communities.	Cross-sectional self-report surveys measuring compliance with non-smoking policies. Part of the Community Intervention Trial for Smoking Cessation (COMMIT) study. Worksites had a 90% response rate, and employed residents had an 80% response rate.	The study examined adherence to workplace smoking policies. The data were collected as part of the Community Intervention Trial on Smoking Cessation (COMMIT), a seven-year randomised community trial conducted in 11 matched pairs of communicates across North America. A goal of the trail was to change the normative environment in which smoking exists; therefore local changes in polices around tobacco use were both encouraged and expected. Worksites were one of	N/A	<u>Worksite</u><br>Policy compliance was greater in worksites with:<br>- Stricter policy restrictions<br>- That did not sell cigarettes on site<br>- That reported excellent relationships between labour and management<br><u>Individuals</u><br>Policy compliance was reported as greater by respondents employed at worksites with:<br>- A smoke-free policy<br>- The presence of a stop smoking program<br>- *Respondents with more than a high school education and who were

				the four channels targeted for intervention.		female also reported greater policy compliance<br>**Compliance with worksite non-smoking policy directly related to the restrictiveness of the policy.** Complete ban results in more compliance than designated areas or no policy.
Stillman, F.A., et al. (2013)<br><br>Beijing, China	Hospitals	**(B)**<br>Physicians	Pre-post surveys. Smoking rates pre- and post-policy implementation, change in knowledge, attitudes and practices among physicians (pre-intervention: n=2835; post-intervention: n=2812 from 7 hospitals), and compliance with policy were assessed. Second hand smoke was estimated by passive sampling of vapour-phase nicotine. Content analysis of hospital's written smoking policy was also conducted.	In 2009 the Ministry of Health of Chine and the Chinese Association on Tobacco Control (CATC) issued a policy document providing details on strategies and measures to ensure that a total smoking ban would be achieved in all buildings and facilities in the health administrative sector and health institutions at all levels by 2011. CATC implemented an intervention to help hospitals develop smoke-free hospitals and educate health care professionals concerning the dangers of second hand smoke and promote cessation.	N/A	**Policy comprehensiveness:** study results suggestive of a relationship between the level of environmental nicotine measured and the policy index score (based on content analysis of their written smoke-free policy guidelines). As the policy score increased, the measurable level of nicotine was lower.
Vardavas, C.I., et al. (2013)<br><br>Greece	Hospitality venues (restaurants, bars, cafes)	**(B)**<br>Venues	Air sampling measurements collected over four waves (baseline and then at 6-month intervals) at venues (n=150 at baseline; n=75 at 2-year follow-up). 445 venue exposure measurements made over 2-yr period. Baseline measurements taken before smoke free legislation was introduced.	In 2010 Greece implemented a non-enforced, nationwide smoke-free legislation	Neither indoor nor outdoor signage was found to reduce indoor SHS concentrations.	**Venue type:** bars and cafes had higher SHS concentrations compared to restaurants across all waves.<br><br>The presence of ashtray or ashtray equivalents (**smoking cues**) were strong determinants of indoor SHS concentrations and legislation breaches.<br><br>Compliance declined over time.
Willemsen, M.C., et al. (2004)<br><br>The Netherlands	Psychiatric setting (psychiatric hospitals, outpatient care institutions,	**(B)**<br>staff, attendants/ nurses, and patients	Cross-sectional self-report survey of random samples of treatment staff (n=540), attendants / nurses (n=306), and patients (n=96) assessing ETS exposure, current	Study examined which smoke-free policies were most common, how they were complied with, the amount of environmental tobacco smoke exposure and beliefs determining support for complete	N/A	**Setting type:** Employee exposure to ETS was highest among inpatient settings (i.e. sheltered homes, followed by psychiatric hospitals, and outpatient settings). Exposure was negatively associated with **having a smoking policy in place**

	sheltered home facilities)		smoking policy, compliance with smoking policy, beliefs about smoking bans. Response rates ranged from 47% (patients) to 79% (attendants).	smoking bans within the Dutch psychiatric setting.		(compared to having no smoking policy), and higher compliance.
Williams, A., et al. (2004)<br><br>New Hampshire, US	Restaurants	**(B)**<br>Restaurant managers or owners	Cross-sectional 22-question telephone survey of restaurant managers or owners (n=400) regarding smoking polies in place, attitudes to smoking policy, compliance, and customer satisfaction. Response rate was 31.4%.	The New Hampshire Indoor Smoking Act (effective 1993): smoking prohibited or restricted to designated smoking areas in enclosed places of public ownership or public access and places of employment. Designated smoking areas must be clearly signed, have a continuous physical barrier or space between smoking and non-smoking sections, and ventilation to minimize ETS. Certain public spaces are exempt, e.g. restaurants with <50 (smoking permitted without restrictions).	Respondents reporting that they had ≥1 customer complaints within the last month regarding restaurant smoking policy were 1.9 times more likely to consider going smoke-free than restaurants that received no complaints.	Restaurant characteristics positively associated with permitting smoking:<br>- selling alcohol (and additionally, having a bar or lounge)<br>- selling tobacco<br>- non fast food restaurants<br>- restaurants whose smoking policy was determined by owner or manager (as opposed to the corporate office)<br><br>Compliance with Indoor Smoking Act was low overall. Of those restaurants that permitted smoking, only 40% were compliant with all four components of the law.
Xiao, D., et al. (2013)<br><br>China	Hospitals	**(B)**<br>Project coordinators & hospital staff	Pre-post study using follow-up surveys of project coordinators (8 senior medical doctors, 33 directors of administration) from 41 hospitals spanning 20 province) and cross-sectional surveys of hospital staff (Survey 1: n=24642; Survey 2: n=24087). Surveys included questions regarding the implementation of the new smoke-free policy.	In 2009 the key national stakeholders agreed to implement a complete smoking ban within all medical and health care systems in China during 2011.<br>Hospitals participating in a pilot of the implementation were asked to appoint a project coordinator from among their senior management staff, establish structures to educate staff and patients about the new standard, ban sales of tobacco products, and ban smoking inside the hospital. Associated requirements included routine monitoring of patients' smoking status and provision of stop smoking advice and treatment.	Hospitals improved significantly in all areas with the exception of no-smoking signage (which was already in place at the time of pre-survey).<br>At the end of the implementation period the policy details that were implemented in all 41 hospitals included:<br>- complete ban on sales of tobacco products<br>- complete ban on smoking indoors<br>- official hospital policy encouraging smoking members of staff to use smoking cessation treatments<br>- disincentives for smoking inside the hospital<br>- financial incentives for departments declared "smoke-free"<br>- all staff asked to advise patients to quit<br>Almost all (33-40) hospitals implemented:<br>- designated outdoor smoking areas<br>- instructed hospital doctors to include stop-smoking advice and an offer of	N/A<br>(Over the implementation period there was a significant decline in smoking prevalence among staff.

					cessation treatment in their routine work<br>- set up stop-smoking clinics that are staffed by doctors, and set up cessation phone lines<br>*Routine recording of smoking status in patient notes was only strategy implemented in minority of hospitals.	
